# Magnetic Relaxation Switching Assay Using IFNα-2b-Conjugated Superparamagnetic Nanoparticles for Anti-Interferon Antibody Detection

**DOI:** 10.3390/bios13060624

**Published:** 2023-06-05

**Authors:** Boris Nikolaev, Ludmila Yakovleva, Viacheslav Fedorov, Natalia Yudintceva, Vyacheslav Ryzhov, Yaroslav Marchenko, Alexander Ischenko, Alexander Zhakhov, Anatoliy Dobrodumov, Stephanie E. Combs, Huile Gao, Maxim Shevtsov

**Affiliations:** 1 Laboratory of Biomedical Nanotechnologies, Institute of Cytology of the Russian Academy of Sciences (RAS), Tikhoretsky Ave., 4, 194064 St. Petersburg, Russiayudintceva@mail.ru (N.Y.); 2 Personalized Medicine Centre, Almazov National Medical Research Centre, Akkuratova Str. 2, 197341 St. Petersburg, Russia; 3 Department of Inorganic Chemistry and Biophysics, Saint-Petersburg State University of Veterinary Medicine, Chernigovskaya Str. 5, 196084 St. Petersburg, Russia; 4 Petersburg Nuclear Physics Institute, National Research Centre “Kurchatov Institute”, 188300 Gatchina, Russia; ryzhov_va@pnpi.nrcki.ru (V.R.);; 5 Laboratory of Hybridoma Technologies, Saint-Petersburg Pasteur Institute, Mira Str. 14, 197101 St. Petersburg, Russia; 6 Department of Nuclear Magnetic Resonance, Institute of Macromolecular Compounds of the Russian Academy of Sciences (RAS), Bolshoi pr. 31, 199004 St. Petersburg, Russia; 7 Department of Radiation Oncology, Technishe Universität München (TUM), Klinikum Rechts der Isar, Ismaninger Str. 22, 81675 Munich, Germany; 8 Key Laboratory of Drug-Targeting and Drug Delivery System of the Education Ministry, West China School of Pharmacy, Sichuan University, Chengdu 610041, China; 9 Laboratory of Biomedical Cell Technologies, Far Eastern Federal University, 690091 Vladivostok, Russia

**Keywords:** interferon, IFNα-2b, anti-INFα-2b antibodies, nanoparticles, SPIONs, magnetic resonance imaging, nanosensor, magnetic relaxation switching assay

## Abstract

Type I interferons, particularly IFNα-2b, play essential roles in eliciting adaptive and innate immune responses, being implicated in the pathogenesis of various diseases, including cancer, and autoimmune and infectious diseases. Therefore, the development of a highly sensitive platform for analysis of either IFNα-2b or anti-IFNα-2b antibodies is of high importance to improve the diagnosis of various pathologies associated with the IFNα-2b disbalance. For evaluation of the anti-IFNα-2b antibody level, we have synthesized superparamagnetic iron oxide nanoparticles (SPIONs) coupled with the recombinant human IFNα-2b protein (SPIONs@IFNα-2b). Employing a magnetic relaxation switching assay (MRSw)-based nanosensor, we detected picomolar concentrations (0.36 pg/mL) of anti-INFα-2b antibodies. The high sensitivity of the real-time antibodies’ detection was ensured by the specificity of immune responses and the maintenance of resonance conditions for water spins by choosing a high-frequency filling of short radio-frequency pulses of the generator. The formation of a complex of the SPIONs@IFNα-2b nanoparticles with the anti-INFα-2b antibodies led to a cascade process of the formation of nanoparticle clusters, which was further enhanced by exposure to a strong (7.1 T) homogenous magnetic field. Obtained magnetic conjugates exhibited high negative MR contrast-enhancing properties (as shown by NMR studies) that were also preserved when particles were administered in vivo. Thus, we observed a 1.2-fold decrease of the T2 relaxation time in the liver following administration of magnetic conjugates as compared to the control. In conclusion, the developed MRSw assay based on SPIONs@IFNα-2b nanoparticles represents an alternative immunological probe for the estimation of anti-IFNα-2b antibodies that could be further employed in clinical studies.

## 1. Introduction

Interferon alpha (IFNα) is a well-known pleiotropic cytokine with useful anti-infection, anti-proliferative and immunomodulatory activities, playing an important role both in adaptive and innate immune responses [1,2,3,4,5,6,7]. IFN is produced primarily by mononuclear phagocytes that are stimulated by bacteria and viruses, and which exert a wide range of immunomodulatory activities. Currently, interferon is approved for the treatment of hepatitis, influenza, and multiple sclerosis [8,9]. Particularly, IFNα-2b, a highly potent cytokine that belongs to the IFNα family, was shown to exert the antiproliferative activity (predominantly via the JAK-STAT pathway), and was approved by The Food and Drug Administration (FDA) for treatment of renal cell cancer, malignant melanoma and hairy cell leukemia [10,11]. Furthermore, IFNα-2b was approved for treatment of other malignancies including Chronic Myelogenous Leukemia, Follicular Lymphoma, AIDS-related Kaposi’s Sarcoma [12,13].

On the other hand, the detection of either circulating IFNα-2b protein or anti-interferon antibodies could be employed as a diagnostic approach in revealing the severity of various diseases such as rheumatoid arthritis, type 1 diabetes, systemic lupus erythematosus, multiple sclerosis [14,15,16,17,18,19]. Thus, as was shown previously, individuals with anti-IFN-I autoantibodies demonstrate an enhanced susceptibility towards viral diseases such as influenza, herpes or COVID-19 as well as side reactions to live-attenuated vaccines [20]. Therefore, the development of a highly sensitive platform for analysis of either IFNα or anti-IFNα antibodies is of high importance to improve the stratification and diagnosis of various pathologies associated with interferon disbalance. One of the approaches could be based on the application of superparamagnetic iron oxide nanoparticles (SPIONs) as a highly sensitive nanoplatform for protein detection [21,22,23,24,25,26].

In the current study we aimed to employ a magnetic relaxation switching assay (MRSw)-based nanosensor for the detection of anti-IFNα-2b antibodies. These nanoplatforms respond to the changes of the transverse relaxation time (T2) of H_2_O molecules due to the processes of aggregation/disaggregation of superparamagnetic nanoparticles induced by the analyte [27,28,29]. In a uniform magnetic field, the antibody-conjugate associates form anisotropic filamentous subunit structures, which are subsequently deposited, falling out of the signal detection region of the RF coil of the spectrometer sensor, which leads to a decrease in the proton relaxation rate. Homogeneous immunoassays based on the principles of switch magnetic resonance for detection of antigen–antibody interactions do not require time-consuming sample purification and rinsing stages prior to analysis, in contrast to sandwich immunoassays [30]. MRSw assays exploit the fact that both nanoparticles (NPs) and larger magnetic particles (MPs) have different transverse relaxation times (T2) in the dispersed and aggregated states. As a rule, T2 decreases with the aggregation of MPs, but in some special cases T2 grows with aggregation. These effects depend on a dephasing (loss of phase coherence) of the proton spin precession in the inhomogeneous magnetic field in the vicinity of MPs [31]. The presence of magnetic clusters of conjugate interferon with its antibody influence the relaxation behavior of protons and can be used for biosensing.

Magnetic NPs characterized by high magnetic moments have priority at low concentrations of analyte in MRSw assays [30]. Induced aggregation antigen–antibody (Ag/Ab) gives rise in improved sensitivity [32]. Field-induced MP aggregation enhances analyte-mediated formation of magnetic aggregates. The presented approach is referred to as magnetic field enhanced biosensing technique for testing interferon-antibody interaction in optically dense media. MRSw assays are similar to aggregation assays using latex particles, red blood cell hemagglutination and antibody reactions with proteins (nephelometry). Unlike nephelometry, MRSws do not depend on optical properties of analyte.

In this study we have synthesized superparamagnetic conjugates with recombinant human IFNα-2b protein (SPIONs@IFNα-2b) and by the use of physico-chemical characterization of the particles, demonstrated the feasibility of detecting anti-IFNα-2b antibodies at picomolar level (0.36 pg/mL) employing MRSw. Subsequently, we showed that SPIONs@IFNα-2b particles, due to their MR contrast-enhancing properties, could be used as negative contrast agents (as was demonstrated in in vivo experiments).

## 2. Materials and Methods

### 2.1. Synthesis and Biochemical Characterization of Superparamagnetic Conjugates

SPIONs were prepared by coprecipitation from Fe^2+^ and Fe^3+^ salt solutions (at pH = 10, 80 °C), as was described previously [33,34]. SPIONs were stabilized by cross-linked dextran coating. The hydrodynamic size and Zeta potential of SPIONs and their conjugates were assessed by dynamic light scattering (DLS) using a Zetasizer Nano (Malvern Instruments, Malvern, UK).

The concentration of iron in the nanosuspension was measured by the thiocyanate method [35,36]. In brief, 0.1 mL of the sample was diluted with 0.30 mL concentrated HNO_3_, kept for 10 min at 80 °C, and then cooled to room temperature. Under this condition, the iron in the samples is dissolved and oxidized to the ferric state. Then, the volume of the sample was adjusted to 2.4 mL with water. The samples were then added to 0.8 mL of a 0.8 M solution of potassium thiocyanate where the Fe (III) formed a red complex with the thiocyanate which could be measured by absorbance at 480 or 575 nm.

The recombinant human interferon alfa-2b (IFNα-2b) (FSUE GosNII OCHB, FMBA of Russia) or monoclonal antibody recognizing IFNα-2b (Cytokine, St. Petersburg, Russia) was coupled to NPs via the carbodiimide linker (N-cyclohexyl-N′[β-N-methyl-morpholino-ethyl]carbodiimide-p-toluenesulfonate). Conjugation of SPIONs with bioligands was carried out in a chemical reactor with a propelled activator and a 500 mL jacket at 20–22 °C. The isolation of the conjugate from the reaction medium was carried out on a Dyna magnet (Invitrogen, Thermo Fisher Scientific, Waltham, MA, USA). In the resulting suspension, the amount of iron (III) was determined by the isothiocyanate method; protein was detected by the Lowry protein assay method. Quantitative determination of human IFNα in the test samples was carried out by enzyme-linked immunosorbent assay using a set of reagents “ELISA-IFN-α” (Cytokine, St. Petersburg, Russia).

Preliminary amination of magnetic NPs with dextran coating was carried out according to the following methodology. Briefly, to 6 mL of nanosuspension (at Fe concentration of 1.5 mg/mL) was added 10 mL of 5 M NaOH and stirred in the reactor for 15 min at 260 rpm. To 5 mL of the resulting suspension was added 0.6 mL of epichlorohydrin in portions of 0.2 mL with an interval of 30 min with stirring in the reactor at 260 rpm. After 1.5 h, 2 mL of 25% NH_4_OH was added to the reaction mixture and stirring was continued for 5 h. The resulting suspension was dialyzed against 200 mL of 0.01 M phosphate buffer solution (PBS) pH 7.4 for 48 h to remove residual NH_4_OH. In the resulting suspension, the amount of Fe^3+^ was determined by the isothiocyanate method (0.3 mg/mL) according to the number of amino groups relative to the amino acid glycine (0.0035 M), which amounted to 12 μm of amino groups per 1 mg of Fe^3+^.

To obtain a covalently bound SPIONs@IFNα-2b conjugate, 3.7 mL of aminated nanosuspension was mixed with 0.9 mL of IFNα-2b (0.22 mg/mL). While stirring in the reactor at 260 rpm, 0.2 mL of an aqueous solution of carbodiimide was added dropwise to the mixture in order to activate the carboxyl groups of IFN for binding with the amino groups of the NPs and stirring continued for 2 h. The prepared suspension was dialyzed against 200 mL of 0.01 M phosphate buffer pH 7.4 for 48 h. After washing twice with PBS, the content of IFNα-2b was determined in one of the sediments by the ELISA method using a set of reagents “ELISA-IFN-α” (Cytokine, St. Petersburg, Russia); in the other, the protein was estimated by the Lowry protein assay and the content of Fe^3+^ by the isothiocyanate method. To eliminate the error in protein determination in a slightly brown colored solution (due to the presence of iron), iron was preliminarily dissolved in 0.1 mL of 24% nitric acid. The excess acid was neutralized with 0.2 mL of 2 M sodium hydroxide. Sequential double washing resulted in the removal of unbound IFNα-2b from the supernatant. The conjugate precipitate obtained after magnetic separation and washing twice was collected after centrifugation at 10,000 rpm for 10 min. In the sediment, the relative content of INF to Fe^3+^ was 120 μg:2370 μg (as measured by ELISA).

### 2.2. NMR Relaxometry Assessment of the Magnetic Conjugates and MRSw

The magnetic iron oxide NPs labeled with IFNα-2b were studied for relaxation behavior under the influence of analyte in close proximity to the magnetic core.

The 1H-NMR spectra and magnetic relaxation times T1, T2 were measured by proton magnetic resonance on a Bruker spectrometer CXP-300 (Bruker, Mannheim, Germany) at the frequency of 300 MHz. To estimate magnetic relaxation times, the inversion recovery and Carr–Purcell–Meiboom–Gill (CPMG) pulse sequences were applied. The dependence of proton relaxation times on the concentration of magnetic NPs in was studied in 5 mm tubes. The coefficients of the relaxation efficiency R2*, R2, R1 (relaxivity) were determined from the slopes of the concentration plots of the inverse times of the magnetic relaxation.

For analysis of SPIONs@IFNα-2b coupling with soluble anti-IFNα-2b antibodies the magnetic conjugates or non-conjugated particles (Fe concentration of 150 µg/mL) were co-incubated with antibodies for 4 h. The evaluation of the specific ability of SPIONs@IFNα-2b conjugates to bind antibodies was performed by temporal measurement of the T2 relaxation times of the conjugate suspension in water. This method was previously reported by Tassa et al. for detection of antigens with a sensitivity comparable to ELISA [37].

The gel phantom MR images of magnetic conjugates embedded into a 1% agarose gel were registered with the help of the high-field (11 T) Bruker Avance ΙΙ NMR spectrometer (Bruker, Mannheim, Germany). The measurements were carried out employing multiscan-multiecho (Carr-Purcell) (MSME) sequence (Echo time (TE) = 10 ms, Repetition time (TR) = 2000.0 ms, NAverages (NA) = 3, Echo Number. (EN) = 16); gradient-echo sequence (GEFI ORTO) (TE = 2.4 ms, TR = 100 ms, NAverages = 5, RareFactor = 4); RARE-T1–T1-weighted scan (TE = 7.5 ms, TR = 1300.0 ms, NA = 5, RareFactor = 4); Turbo-RARE-T2–T2-weighted scan (TE = 12.0 ms, TE = 36.00 ms, TR = 4200.0 ms, NA = 5, RareFactor = 8).

### 2.3. Assessment of the Superparamagnetic State of NPs

To assess the magnetic state of iron oxide magnetic nanoparticles (MNPs), a sensitive method was used to record the longitudinal nonlinear response to a weak variable *ac* magnetic field *h*(*t*) = *h* sin(*ωt*) (frequency *f* = *ω*/2π = 15.7 MHz and *h* = 13.8 Oe). A stationary field H was used in parallel with the *ac* field. This method and a self-made set-up are described in detail in [38]; the set-up was adapted for studying MNPs, as described earlier in [39]. The nonlinear response of the MNP ensemble to the ac field in the presence of a constant field is characterized by asymmetric distortions for the positive and negative signs of *h*(*t*), therefore, predominantly even harmonics of the fundamental frequency will be generated in the sample under study. The signal is recorded at the second harmonic with the largest amplitude (*M*_2_). The ensemble of MNPs should show a strong nonlinearity in the dependence of the magnetization M on the applied magnetic field. Registration of the dependence of *M*_2_ on *H* makes it possible to observe a characteristic signal from MNPs with an extremum in a weak field *H*, the position of which is determined by their magnetic moments. The appearance of an *H* hysteresis in the *M*_2_(*H*) response indicates the presence of regions with a ferromagnetic moment in the sample. For MNPs, the presence of field hysteresis indicates a deviation from the superparamagnetic regime. For an ensemble of noninteracting single-domain NPs, the observation of hysteresis indicates the presence of magnetic anisotropy, which includes the contributions of magnetocrystalline anisotropy, shape anisotropy and surface anisotropy of the NPs. As shown earlier, in this case, the shape of the hysteresis curves depends on the scanning frequency and temperature [38], and the superparamagnetic regime is realized above the blocking temperature *T*_B_. The nonlinear response signal can be described by the Bloch equation. If the condition *M*_2_∝*h*^2^ is satisfied, we can introduce the second-order susceptibility:

(1) $$\chi_{2}\left(\omega \right)=\frac{\Gamma }{-2i\omega +\Gamma }\chi^{\left(2\right)}-\frac{\left(\partial /\partial \omega_{0}\right)\Gamma }{\left(-2i\omega +\Gamma \right)\left(-i\omega +\Gamma \right)}\chi^{\left(1\right)}$$

where *χ*^(1)^ = ∂*M*(*H*)/∂*H*, *χ*^(2)^ = (1/2) ∂^2^*M*(*H*)/∂*H*^2^, *ω*_0_ = *gµ*_B_*H*/*ħ*—Larmor frequency, *µ*_B_—Bohr magneton, *Γ*—magnetic relaxation rate. The first term (1) arises due to the nonlinearity of the magnetization curve *M*(*H*) having a static limit, and this term forms the main contribution to the real part of the Re*M*_2_ response. The second term in (1) is due to the influence of the external magnetic field *H* on the relaxation process; it has no static limit and makes the main contribution to the imaginary part of the Im*M*_2_ response, which has the opposite sign with respect to Re*M*_2_ according to Equation (1). For an ensemble of MNPs with a high nonlinearity (due to the saturation of the magnetization in a small constant field), the *χ*^(2)^(*H*) dependence will exhibit an extremum in a weak field (approximately at the inflection point on the *M*(*H*) curve). In the case of uniaxial anisotropy, if we neglect the effect of the field on the relaxation rate of a system of magnetic NPs, *Γ* is described by the expression:

(2) $$\Gamma =1/\tau =f_{0}\exp\left(-E_{B}/kT\right);E_{B}=KV$$

where *V*—particle volume, *K*—anisotropy constant, *k*—Boltzmann’s constant and the frequency *f*_0_ is about 10^9^ s^−1^. For magnetite NPs, the cubic magnetocrystalline anisotropy of the bulk magnetite can be replaced by an effective uniaxial anisotropy, the value of which will be ~12 times smaller for NPs with a shape close to spherical [40]. Thus, the equilibrium state in an ensemble of NPs after switching on the external field *H* will be achieved after a change in the orientation of the moments relative to the anisotropy axis in some part of the ensemble. This is done by setting an energy barrier, which depends mainly on both the anisotropy and the Zeeman energy, so the relaxation rate of this process will be a function of the field. The probability that the magnetization will remain in its original position for time t after the external field is turned off can be written as *P*(*t*) = exp(−*t*/*τ*), which gives, for the residual magnetization, *M*_ret_(*t*)/*M*_0_ = exp(−*t*/*τ*). Thus, if the registration time *t* is less than *τ*, *M*_ret_ will be detected. During periodic *H*-scanning with synchronous recording of the response, a *H*-hysteresis in Re*M*_2_(*H*) will be observed when the period of the *H*-scan is *F*_sc_^−1^ ≤ *τ* = *Γ*^−1^. From this relation, one can find the blocking temperature *T*_B_, below which the *H*-hysteresis will be detected in the dependence *M*(*H*) as well as in the response *M*_2_(*H*). Thus, the mode of magnetic behavior of NPs and its possible transformation can be controlled by the presence of hysteresis *H* in their *M*_2_ response. In our experiments, the condition *M*_2_∝*h*^2^ was satisfied, which ensures the applicability of analytical Equation (1) for the analysis of the experimental data.

### 2.4. Evaluation of Biological Activity of Magnetic Conjugates

The biological activity of IFNα-2b in the samples was determined by the suppression of the cytopathic effect of the vesicular stomatitis virus (VSV) on the cell line of human embryonic fibroblasts L-41. L-41 cells were grown in 96-well flat bottom culture plates to a monolayer. Next, the supernatant was removed from the wells and 100 µL of serial dilutions of the studied analyte preparations were added. A total of 100 μL of serial dilutions of a standard sample of IFN with known biological activity was added to individual wells. After 18 h of co-incubation, the supernatants were removed and VSV (50,000 PFU) in 200 µL of medium was added to the wells. After 24 h, the supernatants were removed and the cells were stained with a solution of 0.5% crystal violet (Sigma) in 20% methanol for 5 min. The plates were then washed with water and dried. Stained cells were lysed with a buffer containing 2% SDS and 5% glycerol in distilled water (100 µL/well) for 10–15 min on a shaker. Optical density was measured at 595 nm on a Victor2 plate photometer (Perkin-Elmer, Shelton, Connecticut, CT, USA). According to the optical density data in the wells, in which the cells were incubated with different concentrations of the standard IFN preparation, a calibration curve was built, according to which the biological activity of IFN in the studied samples was subsequently determined.

### 2.5. Cells

The L-41 cells were obtained from the “Vertebrate cell culture collection” of the Institute of Cytology of the Russian Academy of Sciences (St. Petersburg, Russia; supported by the Ministry of Science and Higher Education of the Russian Federation (Agreement no. 075-15-2021-683)). Cells were grown in DMEM/F12 medium (Gibco, Carlsbad, CA, USA) supplemented with 10% fetal bovine serum (FBS) (Termo Fisher Scientific, Carlsbad, CA, USA) and antibiotic gentamicin 50 μg/mL (Gibco, Carlsbad, CA, USA) at 5% CO_2_ and 37 °C. Before the experiments, L-41 cells were grown in the log phase, viability was determined by 0.4% trypan blue exclusion. For virus infection, cells were cultivated to 80–90% confluence and were infected with wild-type VSV (Institute of Cytology, St. Petersburg, Russia) in DMEM/F12 medium with 10% FBS at a multiplicity of infection of 10 or 0.01 PFU/cell.

### 2.6. Animals

Male 6–8 weeks old Balb/c mice were obtained from an animal nursery “Rappolovo” (St. Petersburg, Russia). All animal experiments were approved by the local ethical committee of the Institute of Cytology (St. Petersburg, Russia) and were in accordance with institutional guidelines for the welfare of animals.

### 2.7. Assessment of the SPIONs@IFNα-2b Accumulation in Intact Animals

The study of the image contrast generated by the introduced conjugate of magnetic NPs with interferon alpha-2b was carried out on Balb/c mice (6 animals per group) as follows: (1) intravenous injection of SPIONs (control) (Fe^3+^ concentration 2.5 mg/kg); (2) intravenous administration of SPIONs@IFNα-2b conjugate (Fe^3+^ concentration 2.5 mg/kg). Twenty-four hours following injection of the NPs, animals were imaged employing the following sequences: spin echo (MSME) (TE = 10 ms, TR = 2000.0 ms, NA = 3, Echo Number = 16) and MSME (T2-map).

### 2.8. Statistics

For the statistical analysis the parametric Student’s *t*-test was employed for the two continuous variables. The significance level was equal to α = 0.05 for all tests. The confidence intervals were at the 95% level. The one-way ANOVA test and the Kruskal–Wallis test were used for comparison of multiple groups which had few observations.

## 3. Results

### 3.1. Physico-Chemical Characterization of Magnetic Conjugates

According to ELISA measurements, the protein contained in the conjugate is interferon, and its amount is 0.2 μg/mL of suspension. The relative protein content in the conjugate is shown in Table 1.

To confirm the conjugation of MPs with IFNα-2b, the electrophoretic mobility was estimated before and after conjugation. Electrophoretic mobility was assessed using particles of SPIONs and SPIONs@IFNα-2b. Ferrocol and fluid MAG-DX (Acrus organics ltd., Moscow, Russia) at a concentration of 1.5 mg/mL were used as a “gold” standard. Electrophoresis was carried out at a maximum current of 150 mA and a maximum voltage of 250 V in a chamber for horizontal electrophoresis SE-1 (Helicon ltd., Moscow, Russia). To deposit the SPION sample directly into the buffer, a magnet was used, which was placed under the electrophoresis chamber. The concentration of agarose (Helicon ltd., Moscow, Russia) was selected to obtain a dense gel in the concentration range from 0.1 to 0.5%. Electrophoresis was carried out for 30 min. The direction of movement in the frontal electrophoresis and the electrophoretic mobility of the samples in the horizontal electrophoresis are presented in Table 2.

The particle charge was determined by the formula:

(3) $$q=\frac{V}{E}6\pi R\eta$$

where q is the charge of a particle of radius R, C; *V* is the velocity of particles, m/s; E is the field strength, V/m; R is the average hydrodynamic radius of the particle, m; η is the viscosity of the aqueous medium during electrophoresis, Pa.

The relative charge of the particles was calculated for their average hydrodynamic radius. The calculation results are presented in Table 3.

**Figure 1:**
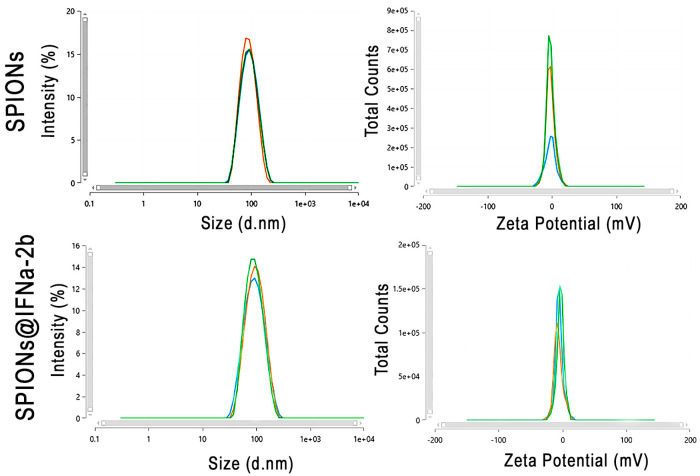
Characterization of the synthesized SPION@IFNa-2b conjugates. Hydrodynamic size and Zeta potential of the SPIONs and SPION@IFNa-2b conjugates. Three lines represent three independent measurements of the sample.

The studied iron oxide NPs coated with dextran had a positive charge at pH = 8.0. The charge of the SPIONs is an order of magnitude smaller than the charge of fluid MAG-DX and Ferrocol MPs. In the process of modification of the SPIONs and the conjugation of IFNα-2b to them, the charge decreased to negative −8.9 × 10^−19^. To further confirm the obtained data the hydrodynamic size and Zeta potential were further evaluated employing the DLS method (Zetasizer Nano, Malvern Pananalytical ltd., Malvern, UK) (Figure 1). Thus, SPIONs constituted 84.9 ± 1.5 nm. Conjugation of IFNa-2b to the NPs increased the size to 87.9 ± 1.5 nm.

### 3.2. Measurement of Aggregate Stability of Hybrid Composites of MNPs during Long-Term Storage

**Figure 2:**
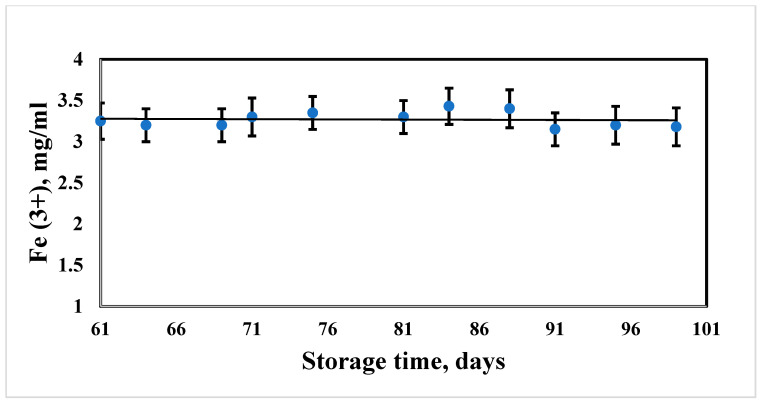
Dependence of the iron concentration in the NP sample on the storage time.

Long-term aggregative stability of the suspensions of hybrid composites of SPIONs is a necessary condition for further application of the particles, either as a contrasting agent or for analytical studies. Measurement of the aggregate stability of the suspensions was carried out by monitoring the total concentration of iron during long-term storage of the laboratory samples. During the study, the suspensions were stored in tightly closed containers (with a volume of 5 mL) in a refrigerator at a temperature of 4 °C. The results of measuring the sedimentation stability and suspension resistance to dilution are presented below (Figure 2). The data are presented in the form of a graphical dependence of the change in the concentration of iron on the storage time. The relative value of the error in measuring the concentration is 7%. The concentration of iron in the suspension after 61 days of storage constituted 3.25 ± 0.22 mg/mL. At day 71, the iron concentration reached the value of 3.3 ± 0.23 mg/mL, then no significant changes were registered until 99 day of storage–3.18 ± 0.23 mg/mL.

### 3.3. Evaluation of the Stability of the Magnetic Properties of Iron Oxide Particles Coated with Dextran

The stability of the magnetic properties of hybrid composites of magnetic NPs coated with 10 kDa dextran was estimated by the dependence of the spin–spin relaxation time of solvent protons (distilled water) on the sample holding time. The sample was stored in a tightly closed ampoule (5 mm) at a temperature of 4 °C. The sample was diluted in a ratio of 1:16, the concentration of Fe^3+^ was 3.0 ± 0.3 mM/L. A spin-echo pulse sequence with multiple echo signals was used to measure spin–spin relaxation times. Thirty-two echo signals were used in the experiment. The duration of the 90° pulse was 4.4 microseconds, the duration of the 180° pulse was 8.8 microseconds. The duration of the pause between 180° pulses was 80 microseconds. Colloidally stable NP samples were examined during 15 days of storage. The criterion for preserving magnetic properties was the constancy of the measured value of the spin–spin relaxation time T2.

**Figure 3:**
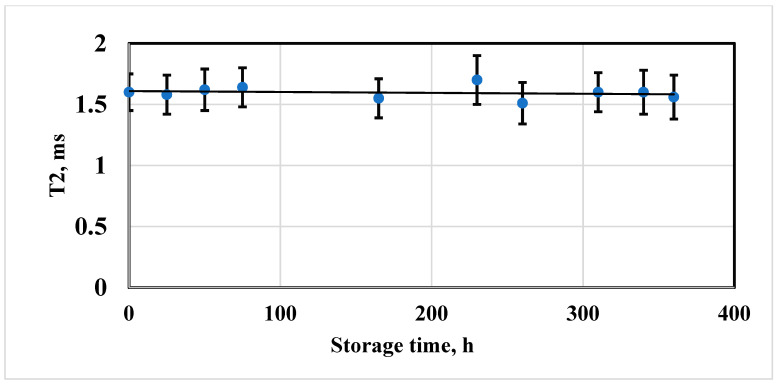
Dependence of the relaxation time T2 of the SPION nanosuspension sample with an iron concentration of 3.0 ± 0.3 mM/L on the storage time.

All aggregatively stable MN suspensions also revealed the constancy of the time value T2. Using the example of the NP sample (Figure 3), a typical dependence of the spin–spin relaxation time of the solvent protons on the storage time is shown. The relaxation time T2 within the observation period varies within the margin of error, which indicates the stability of the magnetic properties. The relaxation time T2 after the first 50 h of storage is estimated as 1.62 ± 0.17 ms. After 115 h of observation there were no changes in the value of the T2 relaxation time (1.55 ± 0.16 ms). At the end of the storage the measured value was registered as 1.56 ± 0.18 ms. Aggregatively stable MN suspensions also maintain stable initial magnetic properties.

### 3.4. Superparamagnetic Properties of the SPIONs@IFNα-2b

**Figure 4:**
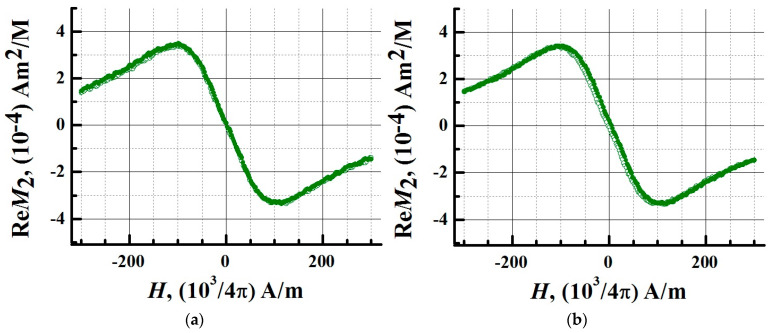
Longitudinal nonlinear response measured at the second harmonic of the *M_2_* magnetization for MNPs. Phase component Re*M_2_* of the second harmonic of magnetization as a function of constant field *H* for aqueous dispersions of MNPs at H-scan frequencies (**a**) 0.25 Hz and (**b**) 8 Hz. The response signals were recorded at a temperature of 294 K. The concentration of iron (in terms of Fe^3+^) in the sample was 0.02 mM/L, the sample volume was 500 μL. Solid symbols denote data obtained during the direct sweep of the *H* field, open symbols denote data obtained during the reverse course of the *H*-scan.

**Figure 5:**
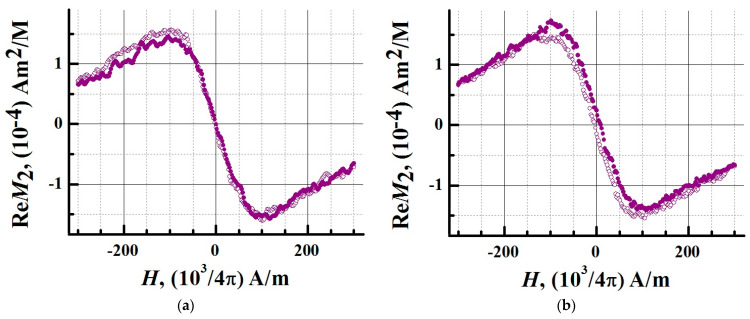
Longitudinal nonlinear response measured at the second harmonic of the *M_2_* magnetization for a bioconjugate of magnetic nanoparticles (MNP-C). Phase component Re*M_2_* of the second harmonic of magnetization as a function of constant field *H* for aqueous dispersions of MNP-C at *H*-scan frequencies (**a**) 0.25 Hz and (**b**) 8 Hz. The response signals were recorded at a temperature of 294 K. The concentration of iron (in terms of Fe^3+^) in the sample was 0.02 mM/L, the sample volume was 500 μL. Solid symbols denote data obtained during the direct sweep of the *H* field, open symbols denote data obtained during the reverse course of the *H*-scan.

In order to determine the magnetic state of iron oxide NPs and their bioconjugate, their aqueous nanosuspensions were studied. The iron concentration in the samples was 0.02 mM/L, the volume of each sample was 500 µL. To detect hysteresis, the signals of the nonlinear response *M_2_* were recorded depending on the field H. The field was scanned linearly symmetrically about the point H = 0 at frequencies of 0.25 and 8 Hz, with an amplitude of 300 Oe. During the experiments, the samples were thermostated at 294 K using evaporated nitrogen. In Figure 4 and Figure 5 the real part of the nonlinear response signals *M_2_* is presented.

At 0.25 Hz, the response signals had no hysteresis. A weak hysteresis appeared at a frequency of 8 Hz (Figure 4 and Figure 5).

The dynamic nature of the hysteresis corresponds to the *M_2_* response of an ensemble of noninteracting MNPs in a single domain state. The absence of hysteresis indicates that the system of NPs under these experimental conditions is in the superparamagnetic regime. Conjugation of NPs with a protein does not change the type of registered *M_2_* signals. At a frequency of 8 Hz, there was a slight discrepancy between the curves of the nonlinear response from the conjugate obtained in the forward and reverse directions of the *H*-scan. At this scanning frequency, the Fe_3_O_4_ system of the magnetic nuclei relaxes with a small delay. The *M_2_* response is provided by the Fe_3_O_4_ nuclei, and an increase in the thickness of the shell of NPs during conjugation does not significantly affect their magnetic parameters. At a scanning frequency of 0.25 Hz, no hysteresis was observed in the nonlinear response signals of MNPs and magnetic conjugates. The method of longitudinal nonlinear response *M_2_* confirmed the superparamagnetic mode of SPIONs and SPIONs@IFN*α*-2b, which generate *M_2_* signals with an extremum in a weak stationary field of ~100 Oe. The study showed that the superparamagnetism of NPs is retained after conjugation.

### 3.5. Determination of the Biological Activity of SPIONs@IFNα-2b

Biological activity was determined in a sample of magnetic NPs labeled with IFN, in which, according to the ELISA data, the protein content constituted 0.2 μg/mL. Non-conjugated magnetic NPs were used as a reference (negative control). The activity of the conjugate when determined in the bioassay for the abolition of the cytopathic effect of VSV on the L-41 cell line was 6000 IU/mL. At the same time, the activity in the preparation of unconjugated NPs was equal to 0. Taking into account the concentration of IFN in the test sample of the conjugate, its specific activity was 3 × 10^7^ IU/mg of protein. The initial specific activity of the IFN preparation used for conjugation was 1.8 × 10^8^ IU/mg. Thus, the specific activity of IFN in the sample of the hybrid magnetic NPs was equal to 17% of the activity of the original protein. Since the studied sample did not contain a non-conjugated protein, the results obtained indicate a partial retention of the ligand activity after conjugation with magnetic NPs.

### 3.6. NMR Characterization of IFNα-2b Magnetic Conjugates

For measurements, a pulsed nuclear magnetic resonance spectrometer CXP-300 (Bruker, Mannheim, Germany), equipped with a cryomagnet containing a vertical cavity, in which a magnetic field of 7.1 T of high uniformity is created, was used. Studies were carried out in standard cylindrical NMR ampoules of 5 mm diameter. Suspensions of SPIONs@IFNα-2b in a volume of 500 μL were used as control samples. To carry out a specific reaction, a solution of monoclonal antibodies against interferon alfa-2b was added to the conjugate suspension. The concentration of antibodies in the resulting suspension was 4 μg/mL, the concentration of SPIONs@IFNα-2b was 0.02 mM/L. The ampoules with the studied magnetic suspensions were introduced by means of an air lift into the region of a uniform magnetic field of 7.1 T of the spectrometer. Measurements of the relaxation times of water protons were performed immediately after the introduction of the ampoule by estimating the time constant of the exponential decay of the nuclear precession. During the measurement, the exponential decay of the echo signals was monitored by the spread of points.

From the moment the sample was placed in the field, the magnetic relaxation times T2 were measured at the proton resonance frequency of 300.13 MHz at 20 °C. The spin-spin relaxation times T2 were measured using a modified CPMG 90°-[τ-180x°-2τ-180-x°-2τ-180x°-2τ-180-x°-τ ]n pulse sequence. The duration of the 90° pulse constituted 5.5 µs, the duration of the 180° pulse was 11 µs, the time τ varied from 800 µs to 2000 µs, the number of echo signals was n = 512.

The study of the relaxation time T2 in the dynamics with a time resolution between exposures of 1 min during the first 10 min and subsequent measurements at long intervals (5, 10, 30 min) provided information on the instability of the structure of the internal field of a suspension of magnetic conjugates, which depends on the aggregation of magnetic nanomarkers in the presence of interferon. The total time of the experiment was 3 h.

Measured at the initial moment (0 min after the introduction of the conjugate suspension to the uniform magnetic field 7 T of the magnet), the relaxation time of water protons T2 was 123 ms. The initial change in T2 of water protons in the magnetic suspension of the conjugate (from 123 to 154 ms) was associated with the fast (1 min) aggregation of magnetic NPs into linear chain structures along the magnetic field lines of the magnet, which led to a redistribution of the spatial structure of the inhomogeneous magnetic field. The order of magnitude of the structural rearrangement time is close to the relaxation times of the reorientation of aggregates of colloidal magnetite particles (10–100 ms) observed in the phenomenon of birefringence when pulsed magnetic and electric fields are applied.

**Figure 6:**
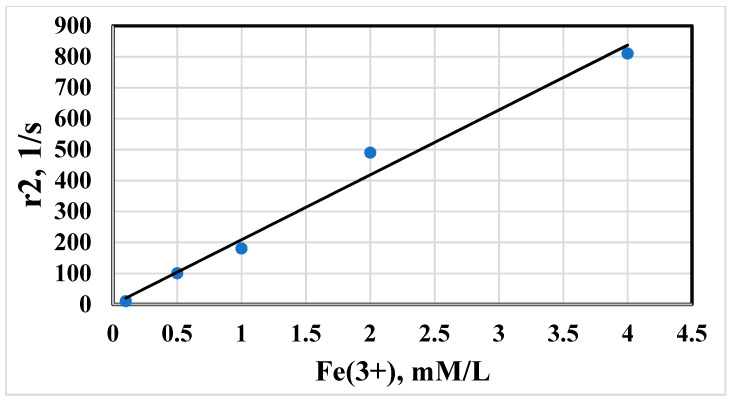
The rate of spin–spin relaxation r2 of water protons in suspension of magnetic iron oxide NPs vs concentration of total Fe.

The proton magnetic relaxation times have been used as criteria regarding magnetism of synthesized NPs. The proton relaxation times T1, T2, T2* of water protons were found to depend on the concentration of particles in suspension. The magnetic relaxation rates r1, r2, r2* as inverse time relaxation values have shown a linear dependence on total Fe content. The experimental results for T2 concentration dependence are shown in Figure 6. The concentration plots followed a linear function:

(4) $$r_{i}=R_{i}C+A$$

where r_i_ = 1/T_i_, i = 1, 2; C = SPION concentration, m; Ri = molar magnetic resonance relaxivity and A = a constant determined by the rate of relaxation of water protons in the absence of a magnetic entity in solution.

The coefficients of magnetic relaxivity Ri were estimated from the slopes of the linear plots. The relaxivity ri of the prepared SPIONs measured at different times is represented in Table 4.

The relaxation efficacy of the SPIONs was significantly higher than paramagnetic ion solutions of Fe^3+^ and corresponded more to the commercial contrast agents Feridex and Ferricol. The magnitude of the magnetic relaxation rate correlates with its appreciable contrast ability. The absence of hysteresis in the particle suspension suggests a superparamagnetic state of the NPs. The increased relaxivity r2 of water protons arises from the time fluctuation of the dipolar interaction between the magnetic moment of the superparamagnetic domain and the proton magnetic moment of the H_2_O. The fluctuating local magnetic field in the vicinity of a magnetic core is governed by outer sphere relaxation mechanism of nonhomogeneous broadening in magnetic suspensions [31]. The conjugation of SPIONs with interferon IFNα-2b slightly decreased the relaxation efficiency of the composed MPs. The rate of magnetic relaxation increased in linear dependence on conjugated SPIONs@ IFNα-2b, as follows from Figure 6. The decrease of relaxation rate in the magnetic conjugate is caused by hindered diffusion of water protons into the magnetic core by the protein coating.

### 3.7. MRSw for the Detection of Anti-IFNα-2b Antibodies

**Figure 7:**
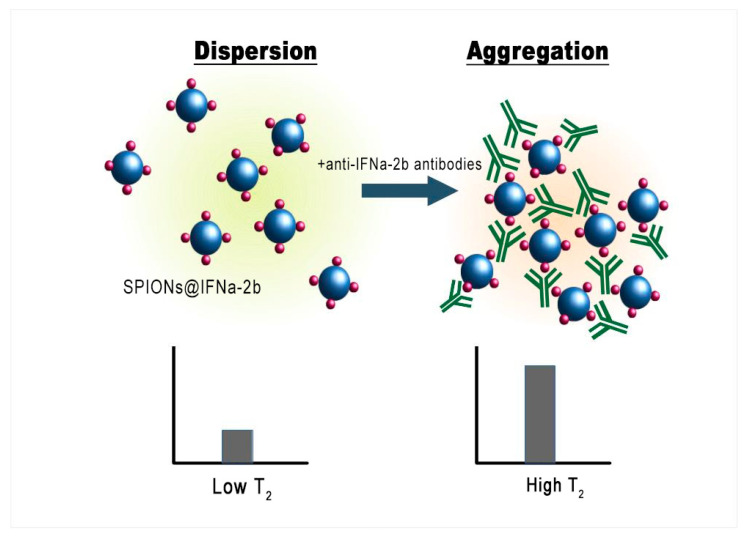
Schematic representation of the MRSw for the detection of anti-IFNα-2b antibodies.

The aggregation of NPs strongly affects the relaxation behavior of water protons in a magnetic fluid (Figure 7).

**Figure 8:**
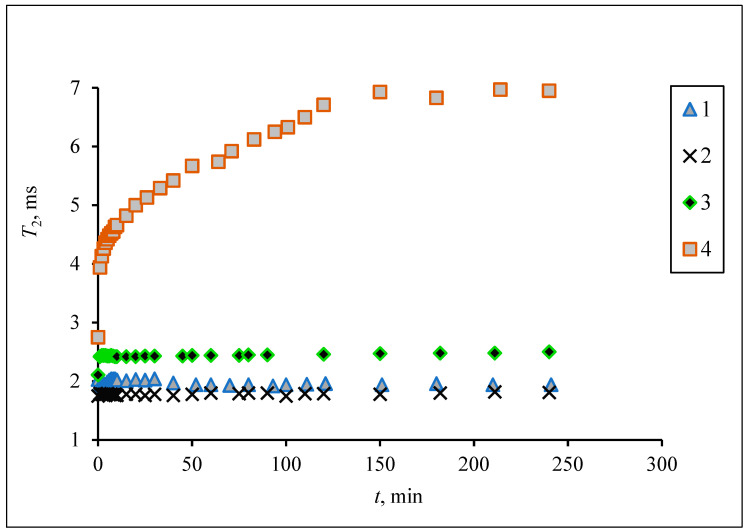
The spin–spin relaxation time of the water proton T2 dependence on the aggregation of magnetic iron oxide NPs of different size fractional dimensions in a homogeneous magnetic field (7.1 T). Fractions prepared by centrifuging at angular speed 12,000 min^−1^ for 5 min (1), 2 min (2), 1 min (3), 0 min (4).

As shown, the synthesized iron oxide NPs have a magnetite core and behave as superparamagnetic substances. Although the prepared superparamagnetic NPs had no initial magnetic remanence, after insertion into the high homogeneous field (7.1 T) of the spectrometer the particles obtained large magnetic moments. Due to dipole–dipole interactions the particles tend to associate, forming aggregates which sediment into the vertical hole of the cryomagnet. Figure 8 demonstrates the growth of the T2 relaxation time of a polysized nanodispersion in the process of removal of large aggregate particles from the resonance detector zone of the spectrometer by gravitational force.

MNPs with a small size have a lower relaxation efficient in comparison with large micron-sized ferromagnetic particles. The IFNα-2b coating induced an additive attraction between NPs, leading to a contribution to the relaxation rate of water protons.

**Figure 9:**
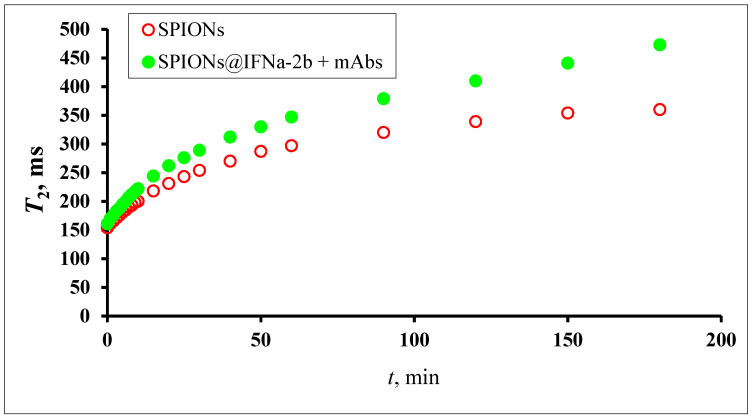
The spin–spin relaxation time of water proton T2 dependence on the aggregation of magnetic iron oxide NPs conjugated with IFNa-2b (0.02 mM) mixed with antibodies (0.36 pg) in a homogeneous magnetic field 7.1 T. MNP fraction prepared by centrifuging at angular speed 12,000 min^−1^.

**Figure 10:**
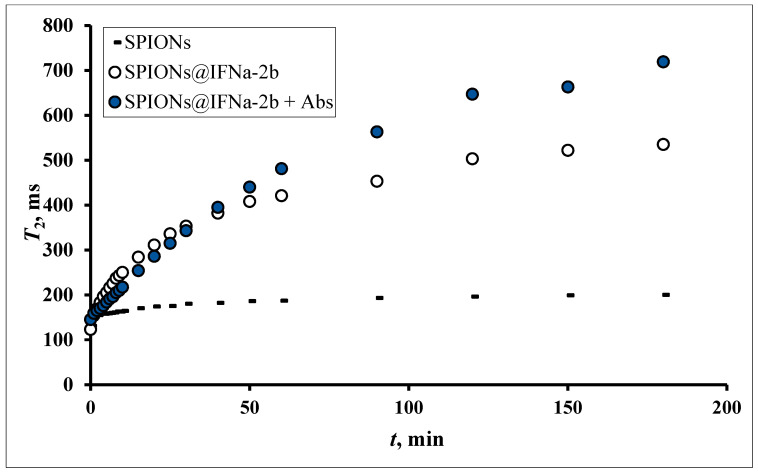
Time dependence of spin–spin relaxation of aqueous protons (T2) on the interaction of SPIONs@IFNα-2b with anti-IFNα-2b antibody.

The addition of an antibody to the magnetic conjugate SPIONs@IFNα-2b changed the time course of the relaxation behavior in a homogeneous field due to specific interaction between the NPs. Being placed in a homogeneous magnetic field, SPIONs modified by an antibody will aggregate. When the interferon binds the antibody the aggregation events are switched on. Particles switch between dispersed and aggregated states, and associated with the change in aggregation are changes in the spin–spin relaxation time (T2) (Figure 9). The aggregated and dispersed forms of MNPs or large MPs have different transverse spin–spin relaxation times T2. NP aggregation and the size range of the resulting clusters depends on the type of analyte and analyte concentration. In the case of conjugated SPIONs@IFNα-2b, the dynamics of the spin–spin relaxation rate is governed by the loss of magnetized clusters from the detection area due to gravitational movement in the course of radio impulse action, see Figure 10. Thus, at the start (0 min) of co-incubation of the NPs with monoclonal anti-IFNα-2b antibodies, the T2 values were 145.5 ± 35.3 ms (non-coated NPs), 123.7 ± 47.1 ms (magnetic conjugates) and 131.3 ± 48.1 ms (SPIONs@IFNα-2b with anti-IFNα-2b antibodies). After 180 min of co-incubation, we observed a nearly sixfold increase of T2 values (719.47 ± 82.1 ms) for SPIONs@IFNα-2b conjugates in the presence of anti-IFNα-2b antibodies due to the formation of aggregates.

**Figure 11:**
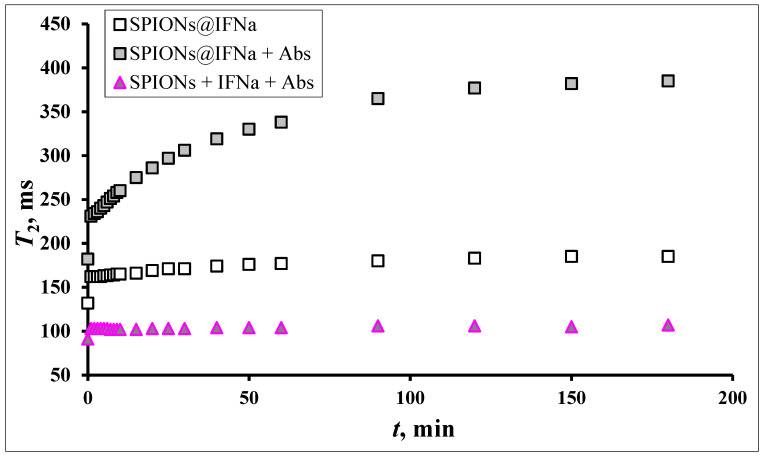
Dynamics of T2 changes depending on the duration of the magnetic field in the reaction of the interaction of SPIONs@ IFNα-2b (0.02 mM) with a polyclonal antibody to IFN (0.2 ng/mL). The onset Ag–Ab reaction immediately before introduction into the magnetic field.

The observed effects of differences in relaxation curves have a specific nature only for conjugated ligands. In the case of a free antibody and interferon in solution the bare MNPs do not sense the presence of proteins, see Figure 11.

Sedimentation of aggregates, which then are no longer “visible” to the resonance detector coil, causes the changes in the relaxation rate evolution. The final result of the analyte-mediated reaction is the formation of clusters with an enhanced magnetic moment which leads to the acceleration of magnetic relaxation of the proton resonance. The functional plots of time relaxation T2 demonstrate the high sensitivity of the NMR switch approach at the level of determination of interferon in solution.

### 3.8. In Vitro Magnetic Contrast Properties of the SPIONs@IFNα-2b Conjugate

**Figure 12:**
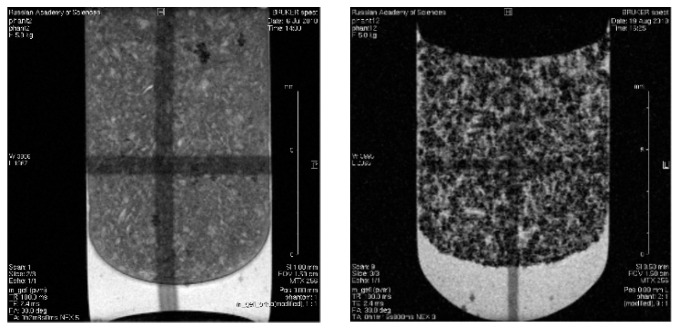
Gradient echo scans in MRI study of agar-agar gel (2% weight) and gel loaded by SPIONs@IFNα-2b. Dark points are the sites of accumulation of aggregated MPs.

An increase in the contrast of MRI images using nanodispersed iron oxide in the composition of interferon conjugates was studied on phantom (model) samples in a 2% agar-agar gel. Phantom samples were prepared in the form of a magnetic gel saturated by NPs coated with dextran filled into ampoules with a diameter of 10 mm. The iron content of the magnetic NPs was 1 mmol/l (0.056 mg/mL). The scans of phantom samples were acquired in gradient echo mode (TE = 2.4 ms, TR = 100 ms). Series of gradient echoes were produced, each of which represents a separately phase-encoded gradient pulse. Control images were generated from blank gel nonloaded by magnetic NPs; Figure 12. The general view of the scans shows that they represent heterogeneous structures. The dark point area evidences the strong relaxation rate induced by distortion of the magnetic field in the vicinity of the magnetic NPs. The bright area in the scans is associated with air bubble formation in the gel. Contrast enhancement was produced by an effective shortening of the spin–spin relaxation time which was found in the linear dependence of the nanoformulated iron content before obtaining T2, T2* measurements in aqueous suspension. Large contrast effects result from cluster formation of the magnetic NPs. According to MR imaging of the phantom sample, the magnetic action of iron oxide NPs is not decreased by the attachment of interferon molecules. The magnetic core is fully responsible for the increase in the relaxation rate. Local inhomogeneities of the field cause strong dephasing of the proton spins due to the high mobility of water around the MPs. Attached interferon does not disturb the magnetic property of the iron oxide nanocrystalline core. Distribution of relaxation times T2* in the plane of the scan confirms the notable sensitivity of the experimental method to the magnetic heterogeneity of the media Supplementary Figure S1.

### 3.9. In Vivo MR-Contrast Enhancing Properties of SPIONs@IFNα-2b Conjugates

**Figure 13:**
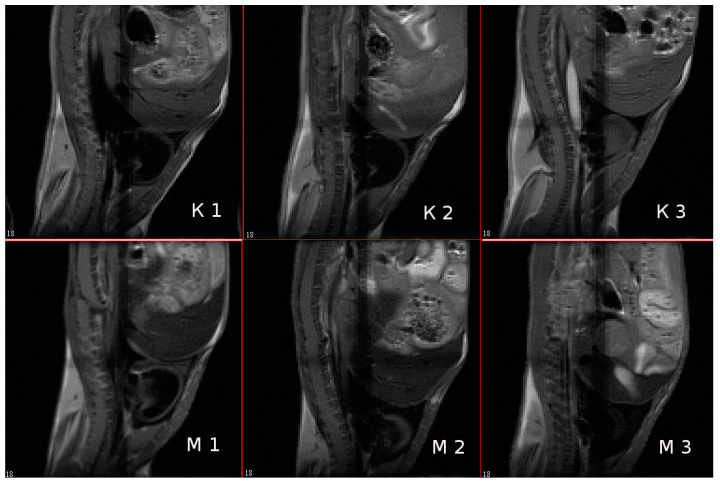
Mouse liver tomograms in sagittal sections (projections). Pulse sequence MSME. TE = 8 ms; (**K1**–**K3**)—control mice, (**M1**–**M3**)—mice 60 min after intravenous injection of 0.1 mL of SPIONs@IFNα-2b conjugate solution (at Fe concentration of 0.01 mg/mL). The numbers in the figures are the numbers of consecutive sections.

**Figure 14:**
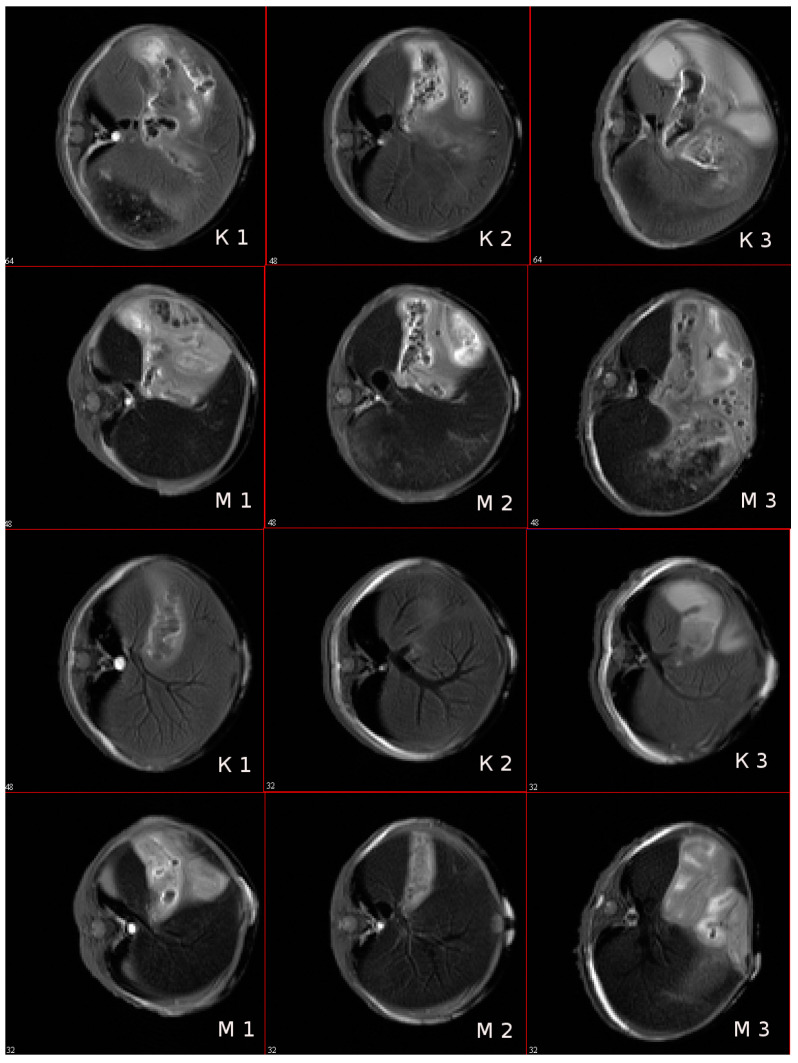
Tomograms of the mouse liver in axial sections (projections). Pulse sequence MSME. TE = 8 ms; (**K1**–**K3**)—control mice, (**M1**–**M3**)—mice 60 min after intravenous injection of 0.1 mL of a solution of SPIONs@IFNα-2b (at Fe concentration of 0.01 mg/mL). The numbers in the figures are the slice numbers.

The ability of magnetic NPs labelled by IFNα-2b to accumulate in tissues in vivo was studied by MR imaging. The targeted entrance of magnetic IFN ensures the growth of rate T2 relaxation accompanied the intensity of the NMR signal in the experiment. The MRI characteristics of the immunoactive magnetic conjugate were detected by a decrease in the optical density of the images of liver tissues after intravenous administration of a solution of magnetic fluid compared with the control, nontreated, mice. The results of a mouse scan in the area of the sagittal and axial projection of the liver, carried out in the MSME pulse sequence mode, are considered with preliminary results on gel systems saturated by MRI, as shown in Figure 12. As follows from the imaging study, the injection of the conjugate leads to a change in the field of darkening of the liver sections and the rate of darkening was similar to the view of the gel phantom in Figure 13 and Figure 14.

A scan time delayed by an hour after the introduction of the conjugate is sufficient to manifest the effect of a negative reduction in the magnetic relaxation times of water protons. Negative contrast is better observed on axial sections of the liver, and the darkening field has a granular structure, see Figure 13. Light areas refer to areas of scanning of organs adjacent to the liver (lungs, stomach, diaphragm and ascending intestines), which, apparently, absorb the contrast agent poorly, or destroy it. The images show the hypotensive network of hepatic veins. The abdominal fat layer in the images gives an increase in the signal due to the increased proton density of the adipose tissue, see Figure 14. To identify a significant effect of the action of the conjugate as a contrasting agent, an analysis of variance was carried out on changes in the average values of the relaxation times T2 of various sections of the liver region before and after intravenous administration of the contrasting magnetic nanosuspension SPIONs@IFNα-2b. The average value of T2 relaxation time of liver tissues in three sections of control mice was 11.6 ± 2.2 ms. Following administration of the contrast agent SPIONs@IFNα-2b, the T2 values decreased to 9.6 ± 2.0 ms. An F-factor of 6.2 and a significance level of 0.019 indicate a significant effect of the contrast agent on the relaxation times of the liver tissues.

An increase in the contrast of the MRI images of the mouse liver following intravenous administration of the magnetic conjugates and the significant change in the T2 relaxation time with intravenous administration at a Fe^3+^ concentration of 0.1 mg per mouse were detected.

## 4. Discussion

Recent decades have shown that the use of nanoscale preparations allows for highly sensitive detection of various chemicals and bioligands (e.g., peptides, proteins, antibodies, viral particles, antibiotics, mRNA, etc.) in biological fluids in real time [41,42,43,44,45,46,47,48,49,50]. MPs were synthesized through the chemical coprecipitation of ferrous (Fe^2+^) and ferric (Fe^3+^) chloride and coated with a 10 kDa dextran via the cross-link with epicholorohydrin. Dextran-coated iron oxide NPs are shown to serve as a versatile platform for targeted molecular imaging and diagnostics using various affinity bioligands [37]. The synthesized magnetic conjugates of SPIONs@IFNα-2b, according to research data, demonstrated a high colloidal stability, magnetic contrast properties, while preserving the biological activity of interferon coupled to the surface of NPs. Thus, according to an in vitro study, SPIONs@IFNα-2b particles had an antiviral effect against cells infected with VSV.

Subsequent studies to evaluate the interaction of the conjugate with monoclonal or polyclonal antibodies directed to interferon also showed a high sensitivity in protein detection in the picomolar concentration range. The employed MRSw provided the ability to detect and quantify antibodies in different sample types, suggesting its clinical utility in the assessment of antibodies in biological fluids (e.g., plasma, serum, etc.) from patients. The ability to sense analyte is observed in both cases of iron oxide NP conjugated with antibody against interferon or coupled with interferon. The affinity in antigen–antibody interaction was independent of the type of bioligand. This interaction leads to a dynamic change of the relaxation rate of water protons in correspondence to theoretical assumptions of diffusion dynamic relaxation [51]. The relaxation efficiency of the instrumental measurement by the NMR method increases due to cluster formation. Magnetic clusters from magnetic conjugates have more magnetic moments to generate a strong inhomogeneous field near associated particles. The presence of stable, visually undetectable, protein clusters of magnetic iron oxide NPs in a strong magnetic field induces additional magnetic interactions that lead to the formation of clusters that are not destroyed by Brownian thermal motion. Clusters have a floccular structure, in which the magnetic cores of particles are part of associates with an effective hydrodynamic size of 1 to 0.1 microns. In a strong homogeneous magnetic field of 7.1 T, the conjugate-antibody associates form anisotropic filamentous subunit structures, which subsequently precipitate, falling out of the detection region of the MNP signal of the RF coil of the spectrometer sensor, which leads to a decrease in the proton relaxation rate, as shown in Figure 5 and Figure 6. Since the field of high homogeneity of the supermagnetic magnet does not exert a magnetophoretic effect on magnetic NPs and their aggregates, the decrease in the effective concentration of magnetic centers along the height of the cylindrical measuring ampoule of the sample should be attributed to the sedimentation of particles in the gravitational field. At a low concentration of particles, hydrodynamic interactions between them can be neglected and the Stokes law can be considered as feasible for NPs and their aggregates. In this approximation, the field-induced coalescence of MNPs into conglomerates due to a random approach will be proportional to the square of the particle concentration, and the magnetic relaxation time T2 increases symbiotically with the average diffusion time around the particle [51]. The reported sensitivity was comparable to the previously published data on the application of MRSw for protein evaluation [30,48,52]. A similar dependence of T2 relaxation of water protons under the influence of antibodies was previously shown for epidermal growth factor and 70 kDa heat shock protein (Hsp70) [53,54].

The measured capacity of the non-coated SPIONs or SPIONs@IFNα-2b conjugates to accelerate the R2 relaxation of water protons (i.e., transverse relaxivity, r2) corresponded to the r2 values of the commercially available agents (Ferrocol) and other reported ferromagnetic particles, thus indicating the MR negative contrast-enhancing properties of the synthesized particles [28,55]. Indeed, subsequent in vivo experiments when SPIONs@IFNα-2b were intravenously administered into intact mice demonstrated that, 24 h following injection, particles accumulated in the liver (as shown on the T2-weighted MR scans). The highest contrast images were achieved using the gradient echo technique and a T2 weighted approach, which revealed a hypointense blackout zone in the areas of the liver in which the accumulation of magnetic NPs occurs when they are captured by Kupffer cells. The scan view after SPIONs@IFNα-2b injection shows signs of increased magnetic heterogeneity and evidence of an increase in the size of hypointense areas on T2-weighted images as a result of accumulation of the magnetic conjugate in the liver. Increased magnetic heterogeneity in liver tissue resembles the scanning view observed in the gel phantom model loaded by magnetic interferon NPs. The pattern of particle retention corresponded to the previously reported data when other functionalized SPIONs were employed [56,57,58,59]. Taking into account the anti-proliferative and anti-viral activity of IFNα-2b obtained magnetic conjugates SPIONs@IFNα-2b could be further employed for the treatment of various pathological conditions. Thus, as was shown by several studies, application of liver-targeted nanocomplexes could be employed for the efficient therapy of hepatitis C virus, human immunodeficiency virus, hepatocellular carcinoma (HCC) [60,61,62,63,64]. Furthermore, magnetic NPs labeled with IFNα-2b with high temporal–spatial resolution and directed towards the inflamed sites of liver parenchyma can be used for differential diagnostics of hepatic disorders [65,66]. SPIONs can increase the detection sensitivity of diffuse hepatic diseases and lesions conspicuity (e.g., cirrhosis, hepatitis, HCC) that are manifested in T2-weighted images in varying degrees of decreased liver signal [67,68,69,70,71]. Thus, as was shown by Park et al. SPIONs-enhanced MRI could help to differentiate the well-differentiated HCCs from mild or poorly differentiated HCCs, particularly on T2*-weighted images [69]. Therefore, MRI study could employ the biosensing ability of SPIONs@IFNα-2b particles to detect inflammatory liver sites as well as other pathologies, although further preclinical studies are required.

## 5. Conclusions

A biosensing system of a magnetic relaxation switch assay based on the superparamagnetic NPs labelled with IFNα-2b can serve as a nanoplatform for a real-time immunoassay of anti-IFNα-2b antibodies. An effect of T2 spin–spin relaxation time change in a homogeneous, high (7.1 T) magnetic field has been demonstrated using a suspension of magnetic conjugates SPIONs@IFNα-2b implemented in Ab–Ag interaction with a picomolar sensitivity of antibody testing at 0.36 pg/mL. Magnetic sensor system has an advantage connected with the possibility of employing this platform with optically non-transparent media. Magnetic interferon conjugate preparation exhibits the properties of a targeted negative contrast agent in MRI diagnostics of liver disorders and can be recommended for further study at the next stages of preclinical trials.

## Figures and Tables

**Table 1:** The composition of the magnetic conjugate of SPIONs with IFNα-2b.

SPIONs@IFNα-2b	**Characteristics**				
	Initial mass ratio [Fe]:[Protein]	Fe^3+^, µg/mL	Protein, µg/mL	Protein/Fe^3+^	Protein, µg/mL (IFA)
	1:0.7	2370	120	0.05	0.2

**Table 2:** Direction of movement and electrophoretic mobility of the studied samples in a field gradient at pH 8.0.

Sample	Direction of Movement (Cathode “−”; Anode “+”)	Electrophoretic Mobility, cm^2^V^−1^s^−1^
Ferrocol	+	4.0 × 10^−4^
fluid MAG–DX	+	3.5 × 10^−5^
SPIONS	−	8.2 × 10^−6^
SPIONs@IFNα-2b	+	6.8 × 10^−6^

**Table 3:** Values of particle charge of different sizes.

Sample	Average Hydrodynamic Radius, nm	Particle Charge, C	Charge per Particle Surface Unit, C/m^2^
SPIONs	53	+9.2 × 10^−19^	+2.6 × 10^−5^
SPIONs@IFNα-2b	62	−8.9 × 10^−19^	−1.9 × 10^−5^
fluid MAG–DX	25	−1.8 × 10^−18^	−2.3 × 10^−4^
Ferrocol	76	−6.5 × 10^−17^	−8.9 × 10^−4^

**Table 4:** Magnetic relaxation rates r2*, r2, r1 in homogeneous magnetic field 7.1 T vs. time t.

t, s	r2*, 1/s	t, s	r2, 1/s	t, s	r1, 1/s
0	1727	0	1250	0	5.00
90	1344	735	1205	420	5.00
1128	1225	1350	1190	1691	4.59
3519	1149	4020	1111	3735	4.48
7380	1112	7620	1042	7950	4.55
11,550	1074	12,110	952	11,805	4.35
67,080	728	68,040	370	67,740	2.08

## Data Availability

The data that support the findings are available from the corresponding author.

## Supplementary Materials

The following supporting information can be downloaded at: https://www.mdpi.com/article/10.3390/bios13060624/s1, Figure S1: Distribution map of relaxation times T2* in the axial section of the gel part of the phantom sample, loaded by SPIONs@IFNα-2b.
