# Supplementary material for: Magnetic Relaxation Switching Assay Using IFNα-2b-Conjugated Superparamagnetic Nanoparticles for Anti-Interferon Antibody Detection
Source: Biosensors (Basel). 2023 Jun 5;13(6):624. doi: 10.3390/bios13060624 (PMC10296438; doi:10.3390/bios13060624)
Supplement: Supplementary file 1 [file biosensors-13-00624-s001.zip › biosensors-2380803-supplementary.pdf]

Supplementary Material

Magnetic Relaxation Switching Assay Using IFNα-2b-Conjugated Superparamagnetic Nanoparticles for Anti-Interferon Antibody Detection

Boris Nikolaev, Ludmila Yakovleva, Viacheslav Fedorov, Natalia Yudintceva, Vyacheslav Ryzhov, Yaroslav Marchenko, Alexander Ischenko, Alexander Zhakhov, Anatoliy Dobrodumov, Stephanie E. Combs, Huile Gao and Maxim Shevtsov

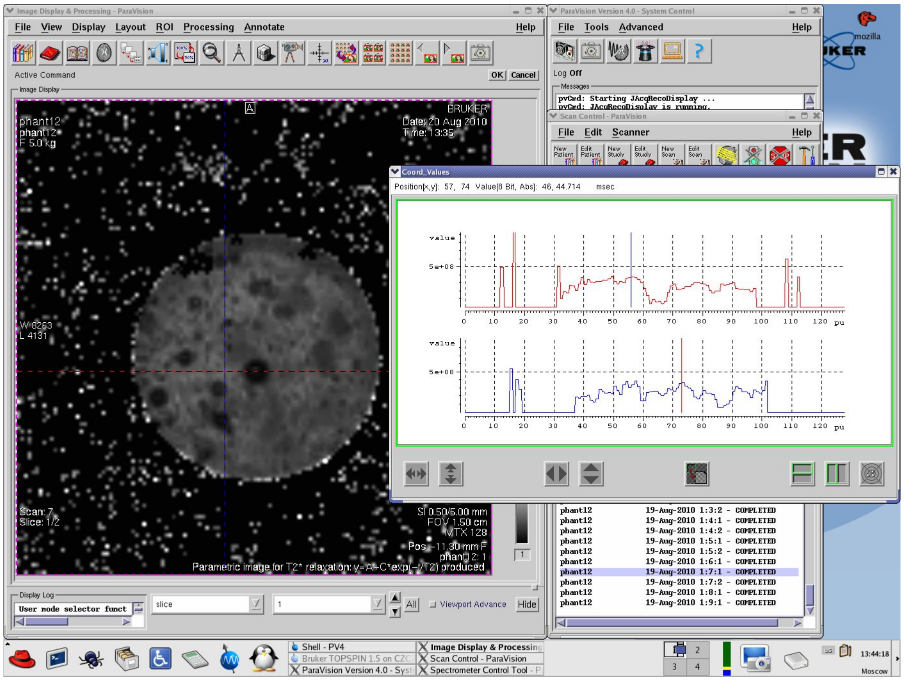

Figure S1. Distribution map of relaxation times T2* in the axial section of the gel part of the phantom sample, loaded by SPIONs@IFNα-2b. The inset to the MRI image shows the change in the relaxation times of the elementary volume along the blue and red lines.
